# Supplementary material for: Are carbon emissions trading and green financial instruments synergistic? -Comprehensive quantitative research based on content analysis
Source: PLoS One. 2024 Mar 7;19(3):e0298601. doi: 10.1371/journal.pone.0298601 (PMC10919601; doi:10.1371/journal.pone.0298601)
Supplement: S1 Data — (ZIP) [file pone.0298601.s001.zip › Data/地级市Z_F工作报告.zip!/╟α║ú/╬≈─■╩╨/└┤╫╘╣1⁄2╓┌║┼í╛┬φ┐╦╩2╛▌═°í┐_2015.docx]

各位代表：

现在，我代表市人民政府，向大会作政府工作报告，请予审议，并请市政协委员和其他列席人员提出意见。

一、2014年工作回顾

2014年，在经济下行压力不断加大的形势下，我们在省委、省政府和市委的坚强领导下，紧紧围绕省委战略任务，团结带领全市各族人民，迎着困难前行，不断开拓创新，奋力打造经济、生态、民生“三个升级版”，较好完成了市十五届人大五次会议确定的目标任务，主要经济指标实现新突破，开创了全市经济社会发展新局面。

——经济运行稳中有进。2014年全市经济发展呈现出增速稳、就业稳、物价稳的良好态势。地区生产总值和固定资产投资首次突破千亿元大关，分别达到1077.1亿元和1176.6亿元，增长13.5%和27.1%。实现地方公共财政预算收入83.9亿元，增长25%，全市总财力突破260亿元。物价过快上涨势头得到有效遏制，居民消费价格累计涨幅2.8%，为近年来最低。固定资产投资、农民人均纯收入、地方公共财政预算收入指标提前一年完成“十二五”规划目标。生产总值、投资、工业增加值、财政预算收入等主要经济指标增速居全国省会城市前列，对全省的辐射和支撑作用进一步增强。

——质量效益稳中有优。实现一产增加值37.8亿元、增长5.4%，二产增加值560.7亿元、增长16.7%，三产增加值478.6亿元、增长9.7%，三次产业比例为3.5:52.1:44.4。全年规模以上轻工业增速高于重工业14.1个百分点，比例由上年的17.7:82.3调整为22.3:77.7。第三产业投资占全社会固定资产投资的比重首次过半，对全市投资增长的贡献率达44.5%。产业结构调整呈现结构优化、转型升级的良好态势。

——民生水平稳中有升。农民人均纯收入突破万元大关，达到10097元，增长12.1%；城镇居民人均可支配收入突破两万元大关，达到21291元，增长9.5%，农民人均纯收入增速高于城镇居民人均可支配收入2.6个百分点。城镇新增就业3.17万人，城镇失业登记率2.98%，完成农村劳动力转移就业41.99万人（次），就业创近年来最好水平。年初承诺的民生实事项目全面兑现，发展成果更好地惠及城乡居民。

一是锐意推进改革，发展动力和活力更加强劲。以改革开放高地来定位、求作为，坚持从制约发展的突出问题改起，重点领域改革全面启动实施，改革效应不断显现。针对行政审批事项过多过繁的实际，加快推进简政放权，共取消下放调整行政审批事项229项，市级审批事项由原来的342项减少到130项，审批事项在西北省会城市最少。建设项目审批时限由近200个工作日缩短到44个工作日。在全国省会城市率先实施了工业投资“负面清单”制度和涉企收费清单制度。建设市公共资源交易中心。针对培育市场主体的需要，深入推进工商登记制度改革，全市各类市场主体达到11万余家，比上年增长2万余家，其中，全年新登记各类企业6222户，注册资本423亿元，分别增长76%和398%，极大激发了市场活力。针对促进投资多元化的需要，有效激活民间投资，开展政府购买公共服务，创新社会投资方式，鼓励引导社会资本参与基础设施和公用事业建设，民间投资占固定资产投资的比重达到58%，成为投资增长的重要支撑力量。针对促进城乡统筹发展的需要，加快推进户籍制度改革，有序推进农业转移人口市民化，提前完成52.5万农业人口转户工作，全市城镇人口比例达72.3%，城镇化稳步推进。针对促进对外开放的需要，把招商引资作为扩大开放的重中之重，服务“青洽会”、举办“城洽会”，签约项目和金额实现双丰收。积极融入国家“一带一路”倡议，新缔结3对国际友好合作城市。西宁国际航空口岸建成开通，我市对外开放进入新阶段。成功引进一批国内外知名企业入驻西宁，全年招商引资到位资金比上年增长30%，再创历史新高。

二是着力优化结构，产业转型升级更加提速。认真贯彻落实促进产业发展的各项政策措施，下功夫调结构、转方式、促转型，努力推动一、二、三产协同发展。着力推进现代农业增量提质。统筹加强基础设施、科技推广、服务体系建设和新型经营组织培育。持续实施“菜篮子”工程提质增效行动，大力推进设施农业建设，积极建设现代农业示范园区，蔬菜自给率有新提升。着力推进工业经济加快转型。针对市场需求不足、工业产品价格回落、企业亏损面扩大等困难问题，采取有效措施努力化解下行压力。依托园区优化工业结构，深入推进两个千亿元、三个五百亿元产业基地建设，完成工业投资478亿元，新增规模以上工业企业25户，工业增加值增长17.3%。全市专利申请量突破千件大关，同比增长40.1%。西宁经济技术开发区在转型升级中实现稳增长，循环经济和结构调整取得新进步，全年技工贸收入、规模以上工业增加值均保持了20%以上增幅，工业总产值首次突破千亿元大关，对全市经济的支撑度有新提升。着力推进服务业集聚发展。积极推进国家服务业综合改革试点，推进建国综合商务区、鲁沙尔文化旅游融合区等服务业集聚区建设。第三产业完成投资601亿元，占全市投资的51.1%。“新三板”挂牌实现零突破。全年实现社会消费品零售总额412.9亿元，增长13.3%，人均消费接近两万元。电子商务等新业态发展势头强劲，建成一批本土电子商务平台。着力推进旅游业加快发展。“清凉西宁”旅游品牌知名度和游客满意度进一步提升。全年接待国内外游客1443.3万人（次），增长10.4%，实现旅游总收入126.8亿元，增长25.9%。

三是全力保护生态，宜居舒适环境更加彰显。落实“生态保护第一”要求，综合施策，标本兼治，大力改善生态环境。加大力度治气。投入资金近14亿元，比上年增长150%。认真开展拉网式大检查，推进大气污染综合治理，实施112个工业企业大气污染治理项目，查封取缔215户扬尘污染严重的煤炭经营户。淘汰报废黄标车1.65万辆，在全国率先完成了年度淘汰任务。开展禁煤区天然气管网覆盖范围内煤锅炉“清零”行动，治理改造974蒸吨燃煤锅炉，煤改气工作在全国大气污染防控重点城市中处于领先。全年空气质量优良率为72.8%，较上年提高12.25个百分点，PM10浓度下降26.8%，在西北省会城市中下降幅度最大。西宁大气环境质量提升至西北省会城市先进水平。多措并举治水。推进湟水河（西宁段）污染治理，湟水河及其一级支流河道非法采洗砂场全部关闭。启动实施总投资15.4亿元的西宁环境综合治理利用世行贷款项目，投资2.31亿元建成第四、第五污水处理厂，投资2.27亿元建成16.2公里城市排水箱涵，投资1.44亿元的再生水项目全面投入运营，湟水河水质整体趋于好转。全面实施北川河核心区综合治理工程，泄洪河道通水，景观水系内河工程六大湖面完成90%的工程量。高度重视水源地保护工作，划定23个集中式饮用水源地保护区实施重点监管。全力以赴增绿。推进国家森林城市创建，完成重点林业工程造林34.5万亩，“四边”绿化7万亩，新增园林绿地194公顷。制定出台《西宁北山美丽园永久性绿地管理办法》，6000亩绿化建设任务提前完成。全市森林覆盖率达32%，建成区绿化覆盖率达40%，人均公园绿地面积12平方米，提前一年完成创森25项核心指标,为西宁申报国家森林城市奠定了坚实基础。注重实效减排。加大工业企业环境监管力度，加快推进钢铁、水泥、铁合金等行业污染设施提标改造，对污染严重、能耗高的企业进行关、停、改措施，实施了电解铝变频技术应用、铁合金余热发电等一批重点节能减排项目。全社会能源消费总量增速控制在4.9%以内，为近年来最好成效。

四是注重建管并举，城市载体服务功能更加完善。把推进城镇化作为从根本上缩小城乡区域差距的重要抓手，重规划，抓管理，强基础。强化规划管理。在深入调研基础上报请省政府印发了加强西宁城市规划建设管理工作的意见，编制完成了《东部城市群西宁都市区战略规划》、《西宁新区总体规划》及九个片区控制性详规等规划，主城区控规编制总面积达100.4平方公里。结合城市精细化建设与管理，开展国家“公交都市”创建工作，投放电召出租150辆，优化城乡公交线路46条。加强重点路段、重点时段交通秩序整治，进一步规范出租车营运和停车场管理。加大投资建设。建国以来西宁地区投资规模最大的市政工程西宁火车站改造历时5年，在全市各有关方面的艰苦努力、不懈奋斗和各族群众的支持中建成运行，西宁进入了高铁时代，广大干部群众攻坚克难、无私奉献、功不可没！围绕东部城市群、基础设施、生态环保、教育卫生等领域，实施各类重点建设项目90项，完成投资347.65亿元。中心广场北扩、南川河综合治理、海湖新区商业核心区等一批重点项目加快推进。投资1.3亿元实施了打通断头路、建设人行过街天桥等39项畅通交通工程项目。城市亮化水平有新提升，基础设施建设力度不断加大，完成投资254.2亿元，增长57.5%，实现了高速增长。开展环境整治。大力推进重点片区征地拆迁工作，依法完成拆迁299万平方米，拆除违法建筑42.7万平方米。城市管理水平进一步提升，渣土运输车辆管理走在西北省会城市前列。以“美丽城镇”和“美丽乡村”建设为牵引，加快城乡一体化建设，“美丽城镇”建设项目顺利推进，58个“美丽乡村”建设任务全面完成，城乡面貌明显改善。

五是高度关注民生，人民生活福祉更加增进。全年民生支出突破200亿元，占全市财政总支出的80%以上，有力保障和改善了民生。狠抓就业创业。坚持把就业创业作为民生工作的重中之重，出台促进就业创业政策措施，加大就业技能培训，建成投用20家创业孵化基地，创业培训2500人，创业带动就业8059人，发放创业小额担保贷款9500万元，进一步激发了全民创业热情。各类失业人员实现就业2.73万人。实施大学生就业促进计划，应往届高校毕业生登记就业率达90.2%。狠抓民生保障。社会保障和就业财政支出28亿元，增长23.8%。提高城乡低保标准，建立低收入家庭经济状况核对机制，推进城乡低保有进有退、动态管理，为困难群众发放最低生活保障金2.13亿元。完善残疾人保障和社会救助制度，继续扩大保险覆盖面，推行农村幸福院建设，市区实现居家养老服务设施全覆盖。筹集17.7亿元资金助推保障性安居工程，开工建设各类保障性住房47790套，实施农村危房改造5157户、农村奖励性住房10021户，为住房困难群众发放廉租房租赁补贴9924万元。保障性安居工程建设力度为近年来最大，走在了全省前列。狠抓社会事业。教育财政支出达34.5亿元，增长12.4%。推行“阳光招生”及素质教育，教育公平得到更好体现。投资19.7亿元，大力实施教育布局调整、标准化学校建设等190个项目，教育惠民政策全面落实。西宁城市职业技术学院开办运行。医疗卫生和计划生育财政支出18.3亿元，增长14.4%。深入推进医改，分级诊疗、双向转诊制度全面实施，城乡居民医保人均筹资标准提高到550元，医保体系更加完善。全市人口自然增长率控制在8‰以内。加快推进重点文化体育基础设施建设，文体事业健康发展，公共服务能力有新提升。狠抓物价稳控。抓好农副产品平价销售网点、平价商店和肉菜直通车建设，加大蔬菜调运储备。继续实行批零差率控制、政府补贴、重点户培育、早市最高限价、群众义务监督员等制度，全面加强市场监管，严厉打击各类价格违法行为，有效抑制了物价涨幅。狠抓扶贫攻坚。大力实施产业扶贫、金融扶贫、雨露计划培训项目。因地制宜支持贫困农牧民种植、养殖和外出务工，实施43个贫困村易地搬迁、86个村整村推进项目，减少贫困人口5.2万人。狠抓公共安全。扎实开展法治西宁、平安西宁、法治政府建设，深入推进民族团结进步先进区创建。强化“大安全”工作理念，统筹社会安全、生产安全、食品药品安全、校园安全等工作，不断提升城市治理能力和水平。及时排查化解社会矛盾隐患，严厉打击各类违法犯罪行为，全市刑事案件等破案率有新提升。全年未发生重特大安全事故，人民群众安全感明显增强。

各位代表，过去的一年，我们认真学习贯彻习近平总书记系列重要讲话精神，扎实开展第二批党的群众路线教育实践活动，加强“三基”建设和政府自身建设，狠抓作风转变，各级干部群众立场、群众观念、群众感情进一步增强。大力推进依法行政，扩大政务公开，加强审计监督、行政监察，严格控制一般性支出和“三公”经费支出。不断提高政府决策和行政透明度，自觉接受人大法律监督、工作监督和政协民主监督，广泛听取各民主党派、工商联和无党派人士意见建议，办理人大代表建议和政协委员提案315件。政府公信力和执行力得到进一步提高。同时，推进军民融合深度发展，国防动员和后备力量建设取得新进步。民族宗教、外事侨务、双拥、气象、工青妇、慈善、残疾人、红十字等工作全面发展。

各位代表，回顾2014年，每项工作都凝聚了汗水和奋斗，每个数字都体现了为民情怀和发展豪情。我们深刻体会到，一切工作都是为了人民，一切工作都要依靠人民。为人民谋福祉永无止境；按规律抓发展至关重要；敢担当抓落实不可懈怠。过去一年工作取得的成绩，是省委、省政府和市委坚强领导的结果，是全市各族人民艰苦奋斗与开拓进取的结果。在此，我代表市人民政府，向全市各族干部群众，向人大代表、政协委员、各民主党派、工商联、无党派人士和各人民团体，向人民解放军驻宁部队指战员、武警官兵、公安干警和驻宁企业，向所有关心、支持西宁建设和发展的同志们、朋友们，致以崇高的敬意和衷心的感谢！

我们也清醒认识到经济发展新常态下面临的特殊困难和挑战，主要是：工业企业经济效益持续下滑，市场主体核心竞争力和抗风险能力不强，转型升级、提质增效任务艰巨；大气和水污染治理、节能减排，淘汰落后产能，水源地保护责任重、压力大，建设生态西宁任务艰巨；城市基础设施还不完善，治理交通拥堵、违章建设、市容环境仍需继续努力，城市承载力亟待提升，城市建设、管理和服务任务艰巨；财政增收压力大，民生改善刚性支出多，扶贫攻坚精准推进，不断提升城乡人民生活质量任务艰巨。此外，行政不作为、慢作为、虚作为等现象还时有发生，干部执行力、落实力需要继续提升。我们要以高度负责的精神，正视问题，解决问题。

二、2015年工作总体部署

今年是全面深化改革的关键之年，是全面推进依法治市的开局之年，也是全面完成“十二五”规划的收官之年，挑战与机遇并存。一方面，经济发展进入新常态，呈现出诸多趋势性变化，矛盾和问题更加显现，宏观经济增长放缓风险加剧；另一方面，国家继续加大对西部地区的支持力度，实施丝绸之路经济带战略，为各项事业发展提供了新机遇，提出了新要求。我们要把握大势，抓住机遇，坚定信心，趋利避害，努力实现新常态下的新作为，不断创造政府工作的新业绩。

今年政府工作的总体要求是：全面贯彻落实党的十八大、十八届三中四中全会、中央经济工作会议精神，深入学习贯彻习近平总书记系列重要讲话精神，按照省委十二届七次、八次全体会议精神和省十二届人大四次会议及市委十三届十次全体会议部署，坚持稳中求进工作总基调，坚持以生态文明理念统领经济社会发展，坚持以提高质量和效益为中心，主动适应经济发展新常态，多措并举稳增长，全力以赴调结构，坚定不移抓改革，更加重视促创新，加大力度惠民生，着力推进市场化、生态化、法治化、人文化、精细化，确保圆满完成“十二五”规划目标，确保经济平稳健康发展，确保社会和谐稳定。

主要预期目标是：地区生产总值增长10%，固定资产投资增长17%，地方公共财政预算收入增长10%，城镇居民人均可支配收入增长10%，农民人均纯收入增长12%，社会消费品零售总额增长12%，居民消费价格涨幅和节能减排控制在省定目标以内，空气质量优良率保持在70%以上。

在经济下行压力持续加大、我市发展基数明显增大的情况下，实现这个目标，存在很大的难度，是一个需要“跳起来”才能够得着的目标，要付出艰苦努力。这个预期目标，体现了省委、省政府对西宁工作的要求，综合考虑了现阶段我市经济增长潜力和今年的宏观经济环境。这些指标都是预期性、指导性的，我们将努力争取更好结果。

为主动适应经济发展新常态，我们本着总体保持稳定、个别适度调整的原则，对“十二五”规划目标进行了合理调整，地区生产总值年均增速由14%调整为13%，第三产业增加值年均增速由12.5%调整为10%，社会消费品零售总额年均增速由15%调整为14%。

做好今年工作，我们将注重把握四个关键。一是牢牢把握稳增长这个总前提。稳增长是解决一切问题的关键和前提。我市经济总量小，发展相对滞后，没有合理的增长速度，就很难为稳就业、增收入提供支撑，就没有民生改善。我们不追求不可持续的高增长，但决不能让增长滑出应有底线，确保经济运行保持在合理区间。二是牢牢把握保护生态这个新要求。“生态保护第一”是我市面向未来发展的战略抉择，我们有责任珍惜和保护好城乡生态。要坚持生态保护优先，守住生态红线，大力推进生态文明建设，有效减少生产生活中的各类污染，增加更多的生态公共产品，走绿色循环低碳发展之路，努力打造宜居宜游宜业宜人的环境，以良好的生态环境吸引投资、服务人民。三是牢牢把握创新驱动这个动力源。实现质量更优、效率更高的发展，必须依靠创新驱动。要加快推进改革，进一步解放思想，破除一切阻碍生产力发展的障碍，在政策的积极引导下，争当改革创新高地，发挥市场力量，优化产业结构，提升产业水平，让市场主体在创新创造中培育新的经济增长点。四是牢牢把握民生福祉这个落脚点。我们最终的目的都是为民谋利。把保障和改善民生放在更加突出位置，集中财力、物力全心全意办好上学、就医、住房、社保、扶贫、公交、文体、养老、安全等与人民群众息息相关的事，让群众更少点怨言，更多些满意，决不辜负人民的期望。

三、2015年主要工作任务

今年，我们将围绕“投资、转型、生态、城镇、服务、民生、改革、法治”八个关键词，着力做好八项工作。

（一）投资是我们的重要支撑，要毫不松懈、优化结构，发挥投资对稳增长的关键作用。依托项目调结构、增后劲、惠民生，深入挖掘投资潜力，着力优化投资结构，推动全市固定资产投资合理较快增长。

发挥重大项目牵引作用。加强项目动态管理，完善重大项目的策划储备、联合审批、跟踪服务、大督查等工作推进机制。认真做好市级重点项目建设，加快西宁轨道交通项目申报等前期工作，完成火车站综合改造项目后续工程，强力推进中心广场北扩、北川片区开发建设、南川片区改造等项目进程，加快完善提升海湖新区，加快规划建设多巴新区。引导房地产及住宅建设合理投资、消费，实施万达广场、城北国际村等建设项目，实现房地产项目投资250亿元以上。

突出项目多元化投资。围绕“三区”战略、产业发展、城中村和危旧房片区改造等重点领域，着力谋划一批调结构、促转型的大项目。引导更多资金投向产业发展、基础设施建设和民生改善领域。加快完善创新招商机制，加强与省外企业的沟通衔接，认真开展194项961亿元筛选储备项目定向招商工作，继续筹办好“城洽会”，实现招商引资到位资金840亿元以上。进一步激发民间投资活力，积极推行政府与社会资本合作模式（PPP），吸引和撬动更多的民间及社会资本参与经济社会建设。积极创新融资方式，规范政府举债行为，推动政府性融资平台企业化经营，在开展中小企业集合债券发行等融资工作的同时，不断拓展直接融资渠道和规模，力争全市重点项目融资达到150亿元以上。

今年要围绕全面建成小康社会总目标，完成“十三五”规划编制工作。观大势、谋大事，集中各方面智慧，正确处理好全局和局部、近期和长远、速度与质量、开放与开发的关系，合理设置目标指标体系，认真研究重大工程、重大项目和重大政策，力争更多项目列入国家和省上重点。

（二）转型是我们的必由之路，要坚定不移、提质增效，提升经济质量和效益。把推动经济提质增效作为主攻方向，注重创新驱动，加快结构调整，做大产业规模，提升产业层次，构建质量好、效益高、可持续的产业体系。

　　推动工业转型升级。把工业作为调结构的主战场，突出项目、融资、服务等关键环节，千方百计扩大总量、优化结构、提升效益，力争工业投资高于上年并有较大增长，工业增加值增长12.5%。进一步发挥开发区支撑全市经济发展的关键作用，支持开发区向特色化高端化发展，大力实施“双百”工程和重大产业基地建设，加快打造新材料、新能源、有色金属精深加工、特色化工、生物医药、藏毯绒纺等产业集群，提升辐射带动能力。扎实推进甘河工业园区循环化改造试点园区建设，抓好一批资源综合利用和产业链延伸项目，在国家循环经济先行区建设上取得实质性进展。

大力发展高效农业。继续加大投入，实施一批农业基础设施建设项目，推动发展方式转变。加快农业产业园区建设，实施集中连片温室改造提升项目，提高设施农业发展水平。着力在蔬菜生产提质增效、优化结构上下功夫，提高冬春季蔬菜生产供给能力。加快发展以饲草为基础的生态畜牧业，新建规模养殖基地45个。大力发展农业产业化，鼓励和扶持种养大户、家庭农场、农民合作社发展多种形式的适度规模经营，全市农民专业合作社发展到2400家。进一步完善农村土地经营权流转政策，抓好湟中县土地承包经营权确权整县推进工作。加强新型职业农民培训，提升农民创业能力。

　　力推服务业提速发展。巩固和扩大国家服务业综合改革试点工作成果，加快改造提升传统服务业，发展壮大新兴业态，力争实现第三产业增加值增长9%。加快服务业集聚区建设，重点推进水井巷中央商务区、绿地中心等大型商贸综合体建设进程。紧紧抓住西宁火车站投入运行的有利时机，大力发展现代物流产业，推动西宁综合保税区建设，不断完善覆盖全省、连接全国的现代速递物流配送体系。推动电子商务发展，鼓励和引导重点商贸流通企业发展网上销售。实施“智慧城市”、4G网络、“宽带乡村”等重点工程。扎实开展全国旅游标准化示范城市创建工作，实施好塔尔寺大景区建设、丹噶尔古城二期开发等重点项目，积极开发精品线路和特色产品，实施乡村旅游富民工程，精心打造“清凉西宁”旅游品牌，力争实现旅游总收入155亿元。

　　增强创新驱动能力。抓好80项国家和省级重大科技项目建设，加快企业技术研发中心、重点实验室等科研创新平台建设，重点在储能电池、轻金属合金材料、装备制造、生物制品等产业实施科技攻关。深入实施千家中小微企业培育工程，加快推进开发区各园区科技创业园、孵化器和西宁中关村科技成果产业化基地及大学科技园建设，加快三县中小企业创业园和城东清真产业园小微企业创业园建设步伐，确保小微企业户数增长25%。坚持人才优先发展，着力培养引进一批领军人才、拔尖人才和紧缺适用人才。努力营造尊重企业家、支持企业家，尊重创业者、支持创新者的良好氛围，全力推动大众创业、万众创新。

（三）生态是我们的最大优势，要坚守底线、肩扛责任，推动生态西宁建设迈上新台阶。以对人民、对历史高度负责的态度，坚持“生态保护第一”，全力推进国家生态文明先行区建设，让西宁的天更蓝、山更绿、水更清。

推进环境综合治理。全力以赴抓创模攻坚，大力实施大气污染防治工程，全面完成天然气管网覆盖范围内燃煤锅炉改造，完成8000辆黄标车淘汰任务。从严控制拆迁和建筑工地扬尘污染，推广绿色节能建筑和低碳环保施工，加强机动车尾气和重点企业污染治理，特别是加快甘河工业园区环保治理项目实施步伐。科学实施人工增雨作业。进一步加强煤炭市场管理，降低煤烟排放，全面改善大气环境质量。大力推进工业窑炉改造、余热余压利用、清洁生产、废气废渣再利用等重点节能工程，力争全市规上工业单位增加值能耗控制在省政府下达的指标范围内。鼓励居民绿色低碳、简约适度消费。

加快生态工程建设。植树造林对于建设生态西宁至关重要。扎实做好国家森林城市创建工作，启动南北山三期绿化工程，继续实施天然林保护、三北防护林等重大生态工程，完成33.39万亩林业生态工程建设和南北山2.3万亩低效林改造。继续推进城区街道绿化、庭院绿化和小游园建设，城区新增园林绿化60公顷，绿化覆盖率达到40.5%，持续不断为生态西宁增绿增美。认真开展好大通、湟中生态文明建设示范工程试点工作。集中连片推进旅游景区、重点干线、中心城镇等区域环境综合整治，全面提高城乡净化、美化、秩序化水平。

持续实施“清水入城”工程。深入实施清洁水行动计划，巩固湟水河污染治理成果，开展重点企业废水深度治理，提高水资源循环利用率。启动西川生态区域规划编制，抓好污水处理厂建设和城区排水管网改造，提升城市生活污水收集率和处理率。全面完成北川河生态河道治理，加快南川河、西川河景观美化亮化，打造“三河六岸”绿色景观生态廊道。启动实施湟水国家湿地公园保护工程，实施好北川、海湖、宁湖等6000亩三大湿地建设，全面开展全国水生态城市试点工作，抓好城市水环境功能区水质达标、饮用水水源地保护工作。

（四）城镇是我们的共同家园，要以人为本、精细管理，以新型城镇化带动城乡一体化。坚持城乡一体、产城融合，高起点规划城市，高水平建设城市，高效能管理城市，促进人口和产业集约集聚，着力提升城市文化内涵和内在品位。

优化城市空间布局。充分发挥规划的宏观指导和建设引领作用，认真开展拓展城市空间发展研究，科学编制《西宁市新型城镇化建设规划》、《城市交通中长期发展规划》、《市域生态基础设施规划》等重点规划。争取并启动实施国家综合管廊、海绵城市试点项目。加大城市基础设施资金投入，重点实施城市地下管线网络智能化建设、城市绿道、供热燃气、四区小城建等一批基础设施项目，不断增强城市基础设施综合服务功能。加快产城融合，以业兴城、以城促业，将开发区各园区建成功能性城区。加快棚户区和城中村改造及新片区建设，实施小桥、杨家巷、北山市场等重点片区开发改造项目。继续加大拆违打非工作力度，有序推进沈家寨、小桥、门源路、曹家寨等重点片区征地拆迁，拓展城市发展空间。加强城市管理，加快实施垃圾无害化处理，抓好户外广告、餐厨垃圾、占道经营等专项治理工作，保持市容和环境卫生长治常洁。年内建成城区百公里自行车绿廊，着力营造绿意盎然的出行环境。

强化城乡基础建设。引导人口向重点城镇集中，加强区域统筹，争取将一批重点建制镇列入国家新型城镇化建设试点。结合三县区位特点和比较优势，大力实施“大县城”发展战略，继续推进“美丽城镇”和“美丽乡村”建设，重点实施大通塔尔、湟中上新庄、湟源大华等12个重点城镇、70个高原美丽乡村等建设项目。精心实施农村环境连片整治项目，做好农村绿化工作。新建农村道路230公里，持续改善城乡居民出行条件。

改善交通出行环境。支持境内京藏高速路建设。通过改革路权，充分利用南北高速路，加快建成内成网络外成环的城区路网，修建西城大街、峡口路与南北高速的连接线。加快世行贷款城市交通项目、湟水路高架、五四西路西延段等项目建设。强力推进“公交都市”创建工作，全面优化公交线网，分批规划建设公交专用道、公交场站和客运枢纽，启动公交智能调度系统建设，引进电动公交运营，推进定制公交、交通高峰期区间公交运行，探索智能交通建设，保障市民出行需求。进一步规范出租车运营市场秩序，建立打击“黑车”长效机制。研究和建立智能信息系统，提高城市停车场管理和服务水平，缓解“停车难”问题。

推动县域经济突破发展。全力推进工业强县战略，加快三县工业园发展，推进特色产业聚集区和生产性服务平台建设，形成推动县域经济发展的新引擎。加大县域经济财政支持力度，在市本级财政预算中安排专项资金支持三县产业发展，拓展融资渠道。统筹引导市区物流、建材、牲畜屠宰等市场向三县适合区域转移，促进城区与县域文化、旅游、商贸、房地产、金融等联动融合发展。

（五）公共服务是我们的重要任务，要补短板、促公平，推动社会事业繁荣发展。坚持底线思维、便民惠民，继续完善提升基本公共服务水平，着力解决群众关注的重点、热点问题，促进城乡基本公共服务均等化发展。

更加注重教育利民。教育既为兴市富民奠基，也为每个人的梦想和幸福奠基。坚持教育优先发展，进一步加大公共财政向教育方面的投入，着力缩小区域、城乡、校际差距。投资15.1亿元实施88项教育重点项目，全面推进三期教育布局结构调整，不断调整优化教育空间布局。加快普及十五年教育，推进义务教育优质均衡发展。深入实施学前教育二期三年行动计划，规范学前教育，提升特色化办学水平。抓好西宁城市职业技术学院建设运行，进一步优化中高等职业教育专业结构。加强教师队伍建设，提高教育教学质量，让每一个孩子都能享受优质公平的教育。

更加注重医疗便民。加快海湖医院、市一医院北川分院、市二医院儿童诊疗中心等项目建设，不断完善医疗卫生设施。积极推进紧密型县乡村卫生服务一体化管理，不断加强医技人员能力培训，构建和谐医患关系，满足基层群众医疗服务需求。发挥红十字会的作用。支持社会资本举办医疗护理、康复理疗、保健养生等医疗机构。进一步完善计划生育利益导向机制，稳妥实施“单独二孩”政策，确保惠民生育政策落到实处。

更加注重文体惠民。以全域视角审视和建设城乡文化，培育和践行社会主义核心价值观，积极开展新一轮全国文明城市创建工作，广泛开展群众性精神文明创建活动。扎实推进国家公共文化服务体系示范区创建工作，抓好基层文化基础设施建设，加快提高公共文化服务能力。挖掘和弘扬地域、民俗文化。创新和提升文化产业，加快河湟民间文化展示中心、藏文化博物馆二期等文化产业项目建设，打造具有本土特色的文化产业品牌。鼓励文化企业开展跨地区、跨行业合作，发展数字出版、网络广播电视、影视制作等新业态。组织开展“百姓大舞台广场演出季”等重点群众文化活动，办好国际徒步节、国际舞蹈节暨“荷花奖”舞蹈大赛等重大文体活动，倡导全民阅读，不断丰富群众文化生活。加强城乡社区群众性文体设施建设，推进公共体育设施免费和低收费开放，让广大群众人人享受运动、人人拥有健康。

更加注重社会治理。加快法治西宁、平安西宁创建步伐，严厉打击各类违法犯罪行为，全力维护人民群众合法权益。依法从严加强安全生产管理，坚决遏制重特大事故发生。推进社会管理重心下移，继续加大社区基础设施建设，积极鼓励社会力量参与社区服务，新建一批社区综合服务中心。强化食品药品监管，确保广大群众饮食用药安全。抓好国防动员和后备力量建设，做好“双拥”工作，不断增强军政、军民团结。支持工青妇工作。深入推进民族团结进步先进区创建活动，依法加强宗教事务管理，全力维护民族团结、宗教和顺、社会稳定的良好局面。

（六）民生是我们得为政之本，要关切群众利益、增进民生福祉，切实提高人民群众生活水平。以更大的决心、更大的力度保障和改善民生，使发展成果更多更公平地惠及城乡居民，让我们的老百姓生活的更加美好、更加幸福。

扩大就业创业。认真落实就业创业扶持政策，鼓励创业带动就业，加大职业培训力度，实现城镇新增就业2.8万人，城镇登记失业率控制在3%以内。积极落实大学生就业促进计划和创业引领计划，高校毕业生登记就业率不低于87%。进一步加强农村劳动力转移就业服务工作，努力增加农民务工收入。加快推进创业孵化基地和创业型社区建设，加大创业贷款和政策扶持力度，促进高校毕业生、城镇困难人员和退役军人就业创业，努力让每一位城乡群众创业有路、就业有岗、实现梦想有机会！

完善社会保障。按照保基本、兜底线的要求，继续扩大养老、医疗、失业、生育等基础保障覆盖面，提高保障层次和水平。进一步健全社会救助体系，继续提高最低生活保障补助标准，规范大病医疗保险制度，真正把保障和救助资金用于救急、济困、解难。不断改善城乡居民住房条件，建设城镇保障性住房40000套、农村危房改造2000户、农村奖励性住房8000户，基本解决城乡困难群众住房问题。支持残疾人和慈善事业。实施养老服务示范基地项目，推进政府购买养老服务试点工作，鼓励社会力量兴办养老服务机构，加大社区居家养老服务中心和农村互助幸福院建设力度，让每一位老人都能安享幸福晚年。

稳控市场物价。坚持建基地、抓流通、管市场并举，继续完善城乡农产品市场体系，规划建设公益性质的标准化农贸市场、直销肉菜店及中小型农副产品批发零售市场。充分发挥平价商店稳价惠民作用，推进蔬菜直销车进社区工作。加大农超对接工作力度，做好蔬菜、肉类储备调运，确保重要商品和物资的有效供给，价格涨幅控制在合理区间。

强化精准扶贫。拓宽产业扶贫，建设一批特色化、专业化产业扶贫基地，完成整村推进项目32个，实施17个村易地搬迁，力争减少贫困人口5.1万人。更加注重智力扶贫，实施职业教育和致富带头人培训工程，提高贫困地区劳动力就业技能，加快改善贫困地区办学条件，决不让贫困一代一代传下去！

（七）改革是我们的最大红利，要与时俱进、攻坚克难，使各项改革举措转化为发展动力。把提高改革质量放到重要位置，继续落实好国家和省上出台的一系列重大改革措施，汇聚起支持改革的正能量，努力在重点领域和关键环节取得突破，使改革红利充分释放，市场活力全面激发。

深化重点领域改革。一是大力推进简政放权。下大气力推动政府自我革命，继续取消下放一批市级行政审批事项，加强事中事后监管，把该放的权力放到位，把该管的事情管到位，力争做行政审批事项最少、审批效率最高、创新创业环境最优的城市之一。加大“权责清单”和“负面清单”制度执行力度。积极稳妥推动公务用车制度改革。二是加快经济体制改革。积极发展混合所有制经济，制定新一轮民营经济倍增计划，着力培育和发展市场主体。稳步推进国有企业改革，鼓励民营资本参股国有资本和交叉持股，加快建成一批混合所有制企业。稳妥推进农村宅基地改革试点。及早运行市公共资源交易中心，统一将全市公共资源交易活动纳入中心交易。全面推进科技项目投入机制改革，实行科技项目分类投入和管理。加强社会诚信体系建设。三是推进财税金融体制改革。完善预算管理制度，全面建立县以上政府涵盖公共财政预算、政府性基金预算、国有资本经营预算和社会保险基金预算的完整政府预算体系。改进政府提供公共服务方式，拓宽政府购买公共服务范围。加快财政专项资金管理改革，集中财力保障重点支出。深入推进金融改革创新，全面激发金融市场活力。四是推进社会事业改革。深化医疗卫生体制改革，全面实施新一轮医改试点重点任务，完善基本药物制度体系建设，统筹推进医疗、医药、医保及支付方式等相关改革。抓好养老服务综合改革试点工作。深化教育改革，启动实施考试招生制度改革，推进“区域教育联盟”管理方式改革试点。加快文化体制机制改革，出台文化小微企业扶持政策。

提升对外开放水平。紧紧抓住国家丝绸之路经济带建设机遇，加强国内外友城合作，充分利用各种重大经贸文化平台，推动对内对外合作交流，力争进出口总额再有较大增长。

（八）为民是我们的履职之要，要顺应人民新期待、恪尽职守，进一步加强政府自身建设。坚持人民性思维，强化群众立场、观点和感情，进一步转变职能、优化环境、改进作风，建设法治政府、效能政府、亲民政府、廉洁政府。

始终坚持依法行政。把依法行政作为基本准则，做到心中有党。坚持在市委领导下工作，主动接受人大依法监督和政协民主监督，提高人大代表建议、意见和政协委员提案办理质量。自觉以法治思维和法治方式管理经济社会事务，完善重大事项集体决策、专家咨询、社会公示、听证评估等制度。做好信访工作。加强“三基”建设。大力推行政务公开，强化行政监督问责，提高依法办事水平，让群众更好地了解政府、监督政府。

始终坚持务实高效。把务实高效作为行政追求，做到心中有责。树立正确政绩观，难事险事敢于担当，大事要事立说立行，崇尚实干，少说多做，低调务实。心态稳定、干劲稳健、工作稳妥，接续奋斗、久久为功。对重大改革、重大项目、重要民生、重要工作列出责任清单，一抓到底，切实落实。坚决做到履行职责不打折扣、兑现承诺不打折扣、服务人民不打折扣！

始终坚持亲民为民。把人民需求作为第一选择，做到心中有民。凡是涉及群众切身利益的决策都充分听取群众意见，凡是有损群众利益的做法都坚决防止和纠正，任何时候都做到不与民争利。尊重规律，顺应形势，把握优势，抓住要事，从人民群众最需要的地方做起，从人民群众最不满意的地方改起，真正做到决策部署依民而定、办法措施应民而出、工程设施为民而建，努力让人民群众享受更多发展成果。

始终坚持廉洁从政。把廉洁自律作为立身之本，做到心中有戒。巩固党的群众路线教育实践活动成果，自觉遵守“三严三实”，认真落实中央“八项规定”、省委省政府21条措施和市委市政府15条措施，从严治政，坚决纠正“四风”。对违法违纪案件有一件查一件，决不搞下不为例，以政府勤政廉政实际成效取信于民。

各位代表，宏伟的蓝图激励着我们，群众的期盼鞭策着我们。让我们更加紧密团结在以习近平同志为总书记的党中央周围，在省委、省政府和市委的坚强领导下，同心同德，真抓实干，为建设人民满意的生活之城、幸福之城，率先在全省实现全面建成小康社会而努力奋斗！
